# Our 12 year experience with Montgomery T-tube in the management of acute blunt laryngotracheal trauma patients

**DOI:** 10.1016/j.bjorl.2020.06.009

**Published:** 2020-07-27

**Authors:** Madhuri Kaintura, Raman Wadhera, Sharad Hernot

**Affiliations:** a Pandit Bhagwat Dayal Post-Graduate Institute of Medical Sciences, Department of ENT, Rohtak, India; b Shri Guru Ram Rai Institute of Medical and Health Sciences, Department of ENT, Dehradun, India

**Keywords:** Acute laryngotracheal trauma, Blunt neck trauma, Montgomery T-tube

## Abstract

**Introduction:**

The Montgomery T-tube is a device used as a combined tracheal stent and tracheostomy tube to prevent post-operative tracheal stenosis.

**Objectives:**

The purpose of this retrospective study is to evaluate the outcome following Montgomery T-tube stenting performed in for neck and airway injury in patients with acute blunt laryngotracheal trauma over a period of 12 years.

**Methods:**

Between 2005 and 2017, 19 patients with acute blunt laryngotracheal trauma underwent Montgomery T-tube stenting. All 19 laryngotracheal trauma patients had undergone a preoperative tracheostomy in the emergency department by an ENT surgeon. Montgomery T-tube stenting was done later through an external approach. The follow up period ranged from 2 to 10 years. The Montgomery T-tube was removed after a period ranging from 6 months to 1½ year.

**Results:**

The majority of patients in the study were in the age group of 21–30 years. A preoperative tracheostomy was done in all 19 patients. All patients except 3 underwent successful decannulation, and experienced long-term satisfactory result.

**Conclusion:**

Management of acute blunt laryngotracheal trauma is a challenging problem that demands a multidisciplinary approach. The ideal treatment option should be individualized according to the patient's condition and characteristics of injury. According to our study we suggest that cases of acute blunt laryngotracheal trauma patients should be managed following the protocol as mentioned in our study, and we strongly emphasize that Montgomery T-tube should be left for at least 1 complete year in the airway as it results in negligible chances of post-traumatic stenosis of airway later.

## Introduction

**Figure 1:**
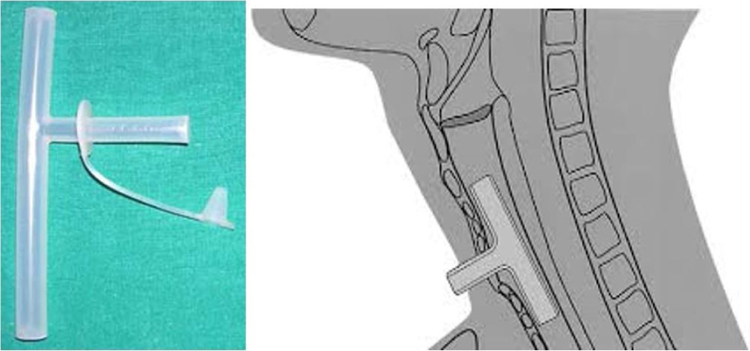
Montgomery T-tube and its placement in the trachea.

**Figure 2:**
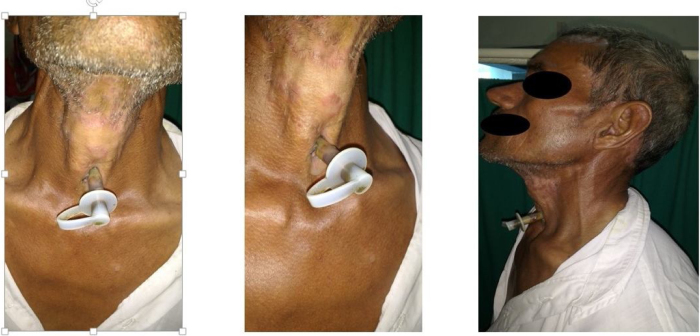
Montgomery T-tube in patient.

The Montgomery T-tube is a device used as a combined tracheal stent and tracheostomy tube to prevent post-operative tracheal stenosis.^1^ Introduced in 1964, the Montgomery T-tube is an uncuffed silicone T-tube that has a long limb which goes into the trachea, and a short limb which protrudes through the tracheostomy stoma (Fig. 1). Montgomery T-tube aids in 2 important ways: (a) It helps in maintaining the patency of the trachea/subglottic, where the lumen has the tendency to progressively narrow because of post traumatic wound contraction, and (b) Assists in reconstructing an adequate airway without the need for a permanent tracheostomy tube with preservation of the vocal function (Fig. 2). Standard tracheal stents like an ETT (endotracheal tube), silastic sheet rolls, and laryngeal stents that can be solid or wound coils,^2^, ^3^ were used previously. A disadvantage of these standard stents was the requirement for an alternative airway in the form of a tracheotomy.^4^ The Montgomery tracheal T-tube has the advantage of being both a stent and tracheotomy tube.^5^

Injury to the larynx or trachea can result in grave airway problems and impaired voice production if not managed promptly. Laryngeal trauma may go unrecognized because patients may appear deceptively normal for several hours after the injury has occurred.^6^ The initial concern with acute laryngeal trauma is securing the airway. Vocal function, which has secondary importance, is often determined by the effectiveness of the initial management.^7^ Controversy exists with respect to the establishment of the airway at presentation, with some favoring intubation^8^ and others preferring tracheostomy.^9^, ^10^ In those who require surgical treatment, the timing of surgery is also controversial. While some recommend early surgical intervention,^10^, ^11^, ^12^ others wait for about four to five days before attempting surgical repair.^13^

Nonetheless, laryngotracheal trauma remains a clinically important injury requiring early recognition, accurate evaluation, and proper treatment. Through the present study, we describe successful airway management using a tracheostomy tube in the immediate post-traumatic period for ventilation to start with, and changing over to Montgomery T-tube as a definitive management for all patients of laryngotracheal trauma. All except 3 patients were eventually decannulated with normal breathing, and dysphonia ranging from minimal to moderate in the long-term.

## Methods

We retrospectively analyzed 19 patients who had undergone Montgomery T-tube insertion post-acute blunt laryngotracheal trauma between 2005 and 2017 after obtaining ethical clearance from the institute vide no. MEEJ/A-IV/2017/1569.

Most of the laryngotracheal injuries in our study fell in 4 broad categories:

Category A: Injury between the level of vocal cords and the lower border of cricoid cartilage, with no damage to tracheal rings (isolated subglottic injury).
Category B: Injury to tracheal rings (isolated tracheal injury).
Category C: Injury to cricoid as well as trachea but not other laryngeal structures (cricotracheal injury).
Category D: Combination of category A, B, and C with damage to other laryngeal structures.

The etiology in these cases was either trauma (roadside accidents, or fall from height), assault and accidental strangulation. All cases on presentation had subcutaneous emphysema present over neck with or without hoarseness of voice. All surgeries were performed by the same surgeon. Preoperative direct laryngoscopy/flexible laryngoscopy was done to confirm the findings of a computed tomography (CT) scan in all patients. These patients were evaluated and grouped according to age, gender, etiology, location and size of the injured segment on CT, follow-up time with Montgomery T-tube, the complications that occurred after T-tube removal and additional tracheal surgery (if required).

We followed a regime in which any acute blunt laryngotracheal trauma was assessed according to the following paradigm:

1) Assessing the patient for subcutaneous emphysema and compromised airway secondary to the laryngotracheal trauma and managing it immediately by doing a tracheostomy at a lower position than normal (i.e. at the level of 3rd/4th tracheal ring).
2) Once airway is secured, patient is shifted to ICU for proper monitoring and routine investigations are sent.
3) Assessing the patient for other major injuries due to trauma.
4) Advising injury-specific radiological investigations (like ultrasonogram [USG] Doppler neck, and contrast enhanced computed tomography [CECT] neck etc.).
5) Finally, definitive surgical management as per the injuries confirmed by the CT scan.

### Technique of Montgomery T-tube insertion

Montgomery T-tube insertion was performed in the operating theater under general anesthesia, usually within 1 week to 10 days of trauma. The ventilation for general anesthesia was given through the tracheostomy tube. As a first step, standard direct laryngoscopy was done to examine the glottic, subglottic, and tracheal region, which sometimes included the passing of a flexible bronchoscope through the direct laryngoscope and inspecting the injured segment closely and clearly. The injured segment was identified and cross-referenced with the findings on CT scan.

**Figure 3:**
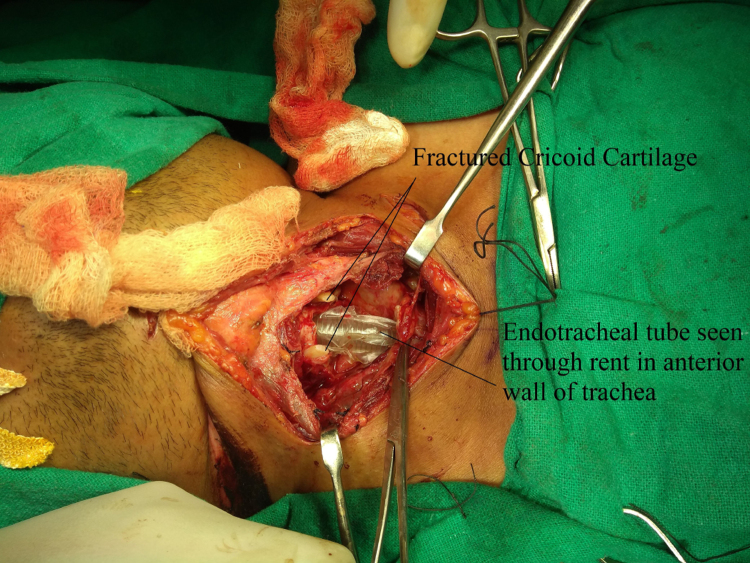
Fractured cricoid cartilage and rent in anterior tracheal wall.

**Figure 4:**
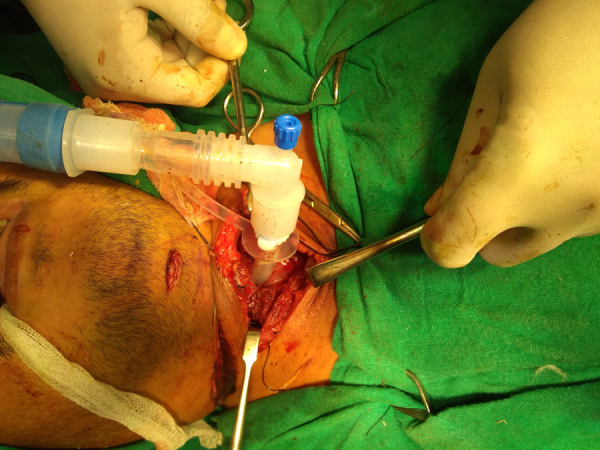
Montgomery T-tube inserted and connected to anesthetic circuit.

**Figure 5:**
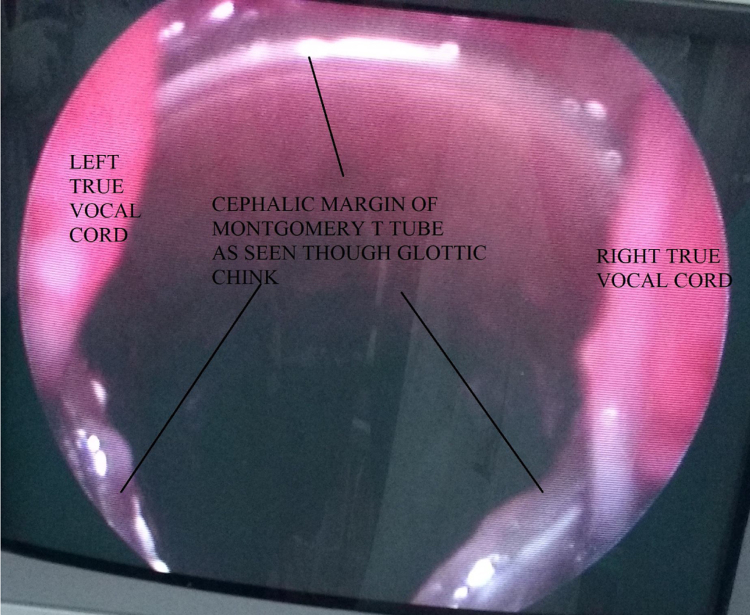
Montgomery T-tube's vertical limb's upper margin as seen through the vocal cords on direct/flexible laryngoscopy.

A horizontal skin crease incision was created at the level of upper border of cricoid cartilage. Subplatysmal flaps were raised. Strap muscles were retracted laterally, and blunt dissection was done to expose the injured area. If present, broken/necrotic pieces of cricoid cartilage and tracheal rings were removed (Fig. 3). The trachea was freed from surrounding structures. Stay sutures were passed from the cricoid cartilage, 1st, 2nd, and 3rd tracheal rings. The anterior tracheal wall was opened, and tracheostomy tube was removed. The Montgomery tube used was of HOOD Laboratories and varied from a minimum 11 mm to a maximum of 13 mm in outer diameter. Size of the Montgomery T-tube was selected appropriately according to the length and location of the injured segment. First the lower limb of M-tube was positioned, and then using artery forceps, the upper limb of M-tube was introduced. The anesthetic circuit was then connected to M-tube (Fig. 4), and laryngeal mask airway was used by the anesthetic team to seal the escape of gases through the open upper end and prevent leakage of anesthetic gases. Using a rigid or a flexible scope the location of upper limb's border was confirmed, so as to make sure that it did not cross the level of vocal cords or touch the undersurface of vocal cords (Fig. 5). Depending on the category of the injury, the trachea was stitched to the cricoid cartilage, and when cricoid cartilage was deficient for suturing, the trachea was directly stitched to the thyroid perichondrium. The anterior tracheal wall was closed over the Montgomery T-tube using sutures or, in some cases, it was reconstructed using septal or conchal cartilage.

The strap muscles were sutured over the repaired segment, and the wound was then sutured in layers after checking for any leak of air using the forced Valsalva maneuver elicited with the help of the anesthetist. Stay sutures were placed from submental region to the chest. A course of broad-spectrum intravenous antibiotics with intravenous painkillers, steroids, proton pump inhibitor was given for a period of 7 days. Regular suctioning was done through the Montgomery T-stent. The skin sutures were removed on the tenth post-operative day, while a follow-up flexible laryngoscopy was performed after a period of 1 month.

The Montgomery T-tube was removed after period varying from 6 to 12 months following complete evaluation.

The patients were followed-up every monthly for first 3–4 months after decannulation for stenosis and/or breathlessness. Long term follow-up varied from 2 to 10 years.

Outcome was assessed in terms of airway and voice. A good outcome was defined as “satisfactory” when the patient had a normal airway or could be decannulated along with a good or fair voice. Patients were asked to evaluate their voice. The following definitions for airway and voice quality were used:

Good airway: Normal airway; patient could be decannulated; patient could do moderate to intense physical activities such as running, climbing 2–3 flight of stairs etc.
Fair airway: Airway narrowing present, but patient could be decannulated; patient could only do light physical activities comfortably and complains of breathlessness on moderate to intense physical activities.
Poor airway: Patient needed a tracheostomy or burial of Montgomery T-tube under the skin for maintenance of airway.
Good voice: No hoarseness or close to pre-injury voice.
Fair voice: Mild to Moderate hoarseness.
Poor voice: Patient cannot raise voice above a whisper.

## Results

A total of 19 patients with ages ranging from 18 to 60 years (median 32.6) formed the basis of this study. There were 15 male and 4 female patients. All of 19 patients incurred injury due to blunt trauma caused by either road side accident, accidental strangulation, assault, or fall from height. None of the admitted patients sustained penetrating injury. Sex distribution of these patients is given in Table 1, which shows that out of 9 road side accidents patients, 8 were males and 1 was female; out of 6 accidental strangulation patients, males and females were 3–3 each; 3 male and 0 female patients had injury due to assault; and 1 male patient received injury due to falling from height. Accidental strangulation occurred due to a loose cloth (muffler/shawl/stole/dupatta) worn around the neck that had got caught in a machine or in the rear tire of a two-wheeler, causing strangulation. Most patients fell in the road side accident group and in the age group of 21–40 years (Table 1). Distributing the patients on the basis of type of injury, 8 patients belonged to Category A injury (i.e. injury between the level of vocal cords and the lower border of cricoid cartilage, with no damage to tracheal rings), 3 patients had Category B injury (i.e. injury to tracheal rings), and 3 patients had Category C injury (i.e. injury to cricoid as well as trachea (aka Cricotracheal injury) without damage to other laryngeal structures), and 5 patients had Combined injury (Table 2).

**Table 1:** Distribution of patients on basis of etiology, gender, and age.

	Road side accident	Accidental strangulation	Assault	Fall from height
*Gender*				
Male	8	3	3	1
Female	1	3	0	0
*Total patients*	9	6	3	1

*Age group (in years)*				
0–20 years	1	1	1	0
21–40 years	4	4	2	0
41–60 years	4	1	0	1
*Total patients*	9	6	3	1

**Table 2:** Distribution of patients on basis of type of injury.

Serial. no.	Category A injury (subglottic injury)		Category B injury (tracheal injury)		Category C injury (cricoid + tracheal injury)		Category D injury (combination of A, B, and C)	
	Male	Female	Male	Female	Male	Female	Male	Female
1.	5	3	2	1	3	0	5	0
Total	8		3		3		5	
Total patients	19							

Emergency tracheostomy was performed in all 19 patients. Hoarseness and dyspnea were found in all patients. Other symptoms suggesting laryngotracheal injury included hemoptysis (9 patients), and odynophagia (9 patients). The cardinal sign of laryngotracheal injury, i.e. subcutaneous emphysema over the neck, and sometimes extending to chest, was present in 16 patients.

In Category C injury patients, 2 patients had crush injury of anterior wall of cricoid and trachea with intact posterior wall, whereas 1 patient had cricoid fracture with injury to the 1st tracheal ring. In Category D injury patients, all 5 patients had undisplaced fractures of the thyroid cartilage, cricoid fracture, tracheal injury with a tear in the cricothyroid membrane. Out of 19, 16 patients had combined soft tissue and cartilaginous injuries (Table 3). All patients underwent surgical treatment. Medical treatment was also given and consisted of voice rest, intravenous steroids, painkillers, antibiotics, and saline nebulization. Surgical treatment in trauma cases consisted of tracheostomy in the immediate post trauma period, where the tracheostomy tube was kept for less than or equal to 10 days in 15 patients, and more than 10 days in 4 patients (Table 3 and 4). A definitive surgical repair in most patients consisting of laryngotracheal exploration, repair, and Montgomery T-tube stenting was done within 10 days (Table 3, Table 4).

**Table 3:** Summary of patients treated for tracheal stenosis or tracheal injury.

Case no.	Sex	Age	Primary disease	Days tracheostomised	Symptoms and signs	Cause of airway compromise	Location of injury	Duration of montgomery t-tube insertion	Complication (long term)	Result
1	Male	34 y	Road side accident	5	Hoarseness + Pain, swelling in neck + Subcutaneous emphysema + Breathing difficulty + Odynophagia	(Category D)	Rent in ant. Wall of trachea with piece of fractured cricoid going in the lumen of trachea + undisplaced fracture of thyroid cartilage + Rent in Cricothyroid membrane + Soft tissue injury of neck	1 year	NIL	Satisfactory
2	Male	20 y	Road side accident	7	Hoarseness + Pain, swelling in neck + Subcutaneous emphysema + Breathing difficulty + Odynophagia	Tracheal injury (Category B)	Injury at 1st and 2nd tracheal ring + Soft tissue injury of neck	6 months	Patient returned with respiratory distress and required retrachesostomy with later reinsertion of Montgomery tube	Unsatisfactory
3	Female	30 y	Accidental strangulation	10	Hoarseness + Pain, Swelling in neck + Subcutaneous emphysema + Breathing difficulty + Odynophagia	Subglottic injury (Category A)	Subglottic injury (undisplaced Fracture of cricoid with rent in cricothyroid membrane) + Soft tissue injury of neck	1 year	NIL	Satisfactory
4	Male	21 y	Road side accident	10	Hoarseness + Pain, swelling in neck + Subcutaneous emphysema + Breathing difficulty	(Category D)	Anterolateral wall of trachea with cricoid fracture + undisplaced fracture of thyroid cartilage + Rent in Cricothyroid membrane + Soft tissue injury of neck	1 and a half year	Peristomal Granulation [dealt with laser]	Satisfactory
5	Male	30 y	Assault	7	Hoarseness + Pain, swelling in neck + Subcutaneous emphysema + Odynophagia + Breathing difficulty + Hemoptysis	(Category D)	Cricoid and 1st tracheal ring fracture with separation and in its anterior part + undisplaced fracture of thyroid + Rent in Cricothyroid membrane + Soft tissue injury of neck	1 year	NIL	satisfactory
6	Female	32 y	Accidental strangulation	14	Hoarseness + Breathing difficulty + Odynophagia	Subglottic injury (Category A)	Subglottic injury (incomplete fracture of cricoid with fracture segment bent toward lumen + cricothyroid membrane rupture) + Soft tissue injury of neck	8 months	Patient returned with respiratory distress and required retrachesostomy with later reinsertion of Montgomery tube	Unsatisfactory
7	Male	25 y	Road side accident	10	Hoarseness + Pain, Swelling in neck + Subcutaneous emphysema + Hemoptysis + Breathing difficulty + Hemoptysis	(Category D)	Cricothyroid membrane rupture + cricoids fracture + 1st tracheal ring injury + undisplaced fracture of thyroid cartilage + Soft tissue injury of neck	1 and a half year	Peristomal Granulation (dealt with laser)	Satisfactory
8	Female	20 y	Accidental strangulation	5	Hoarseness + Pain, Swelling in neck + subcutaneous emphysema + Breathing difficulty + Odynophagia	Subglottic injury (Category A)	Subglottic injury (Crushed injury of cricoid with fracture segment displaced toward airway lumen) + Soft tissue injury of neck	1 year	Mild granulation near repaired segment which was dealt with Flexible bronchoscopy guided Diode laser ablation	Satisfactory
9	Male	25 y	Road side accident	10	Hoarseness + Pain, swelling in neck + Subcutaneous emphysema + Dysphagia + Breathing difficulty + Hemoptysis	Tracheal injury (Category B)	1st and 2nd ring of trachea + Soft tissue injury of neck	1 year 3 months	NIL	Satisfactory
10	Male	35 y	Fall from height	14	Hoarseness + Breathing difficulty + Odynophagia	Subglottic injury (Category A)	Subglottic injury (undisplaced Fracture of cricoid with rent in cricothyroid membrane) + Soft tissue injury of neck	1 year 1 month	NIL	Satisfactory
11	Male	40 y	Accidental strangulation	7	Hoarseness + Pain, Swelling in neck + Subcutaneous emphysema + Breathing difficulty + Hemoptysis	Cricotracheal injury (Category C)	Cricoid fracture with inward protrusion of fractured 1st tracheal ring in the airway + Soft tissue injury of neck	1 year	NIL	satisfactory
12	Male	19 y	Assault	3	Hoarseness + Pain, Swelling in neck + Subcutaneous emphysema + odynophagia + difficulty in breathing + Odynophagia	Subglottic injury (Category A)	Subglottic narrowing due to isolated cricoid fracture + Soft tissue injury of neck	1 year	NIL	Satisfactory
13	Male	24 y	Road side accident	3	Hoarseness + Breathing difficulty + Subcutaneous emphysema in neck + Breathing difficulty + Hemoptysis	Subglottic injury (Category A)	Cricothyroid membrane rent with cricoid fracture + Soft tissue injury of neck	1 year	NIL	Satisfactory
14	Male	30 y	Accidental strangulation	14	Hoarseness + Breathing difficulty + Odynophagia	Subglottic injury (Category A)	Cricothyroid membrane rent with cricoid fracture + Soft tissue injury of neck	1 year 1 month	NIL	Satisfactory
15	Male	22 y	Assault	3	Hoarseness + Pain, Swelling in neck + Subcutaneous emphysema + Odynophagia + Breathing difficulty + Hemoptysis	Cricotracheal injury (Category C)	Cricotracheal separation with intact posterior wall + Soft tissue injury of neck	10 months	Patient returned with respiratory distress and required retrachesostomy with later reinsertion of Montgomery tube	Unsatisfactory
16	Male	34 y	Road side accident	5	Hoarseness + Pain, Swelling in neck + Subcutaneous emphysema + Breathing difficulty + Hemoptysis	(Category D)	Rent in ant. Wall of trachea with piece of fractured cricoid going in the lumen of trachea + undisplaced fracture of thyroid + Cricothyroid membrane rupture + Soft tissue injury of neck	1 year	NIL	Satisfactory
17	Female	35 y	Road side accident	7	Hoarseness + Pain, Swelling in neck + Subcutaneous emphysema + Breathing difficulty + Hemoptysis	Tracheal injury (Category B)	Fracture of 1st two tracheal rings, with necrosis of fractured segments + Soft tissue injury of neck	1 year	NIL	Satisfactory
18	Male	29 y	Accidental strangulation	20	Hoarseness + Pain, Swelling in neck + Subcutaneous emphysema + Breathing difficulty + Odynophagia	Subglottic injury (Category A)	Cricothyroid membrane rent with cricoid fracture with undisplaced facture of inferior thyroid notch of thyroid cartilage + Soft tissue injury of neck	1 year	NIL	Satisfactory
19	Male	33 y	Road side accident	10	Hoarseness + Pain, Swelling in neck + Subcutaneous emphysema + Breathing difficulty + Hemoptysis	Cricotracheal injury (Category C)	Cricotracheal separation with intact posterior wall + Soft tissue injury of neck	1 year	Mild granulation near repaired segment which was dealt with Flexible bronchoscopy guided Diode laser ablation	Satisfactory

**Table 4:** Duration of tracheostomy or endotracheal intubation of patients (in days).

Serial. n°	Duration of tracheostomy (in days)	N° of patients
1	0–5	6
2	6–10	9
3	11–15	3
4	16–20	1
*Total patients*		19

The follow-up period ranged from 2 to 10 years, and no long-term complications were reported in 14 patients. There was no mortality in our study. Almost all patients benefited from the operation in the postoperative period, with relief of respiratory distress and return of phonation.

Sixteen out of 19 patients could be decannulated successfully. Out of 16, 12 patient had a good airway and could engage in moderate to intense physical activities comfortably, whereas 4 patients had a fair airway, who could only do light physical activities comfortably. There were 10 patients who had near-normal voice in the immediate post decannulation period, 5 had fair voice with very mild hoarseness which improved subsequently, 4 had severe hoarseness (Table 5). All these 4 patients had an element of vocal cord immobility, with contralateral vocal cord not being able to compensate, therefore they were referred for speech therapy and kept on long term follow-up. Two patients developed mild granulations near the repaired segment, which were dealt with flexible bronchoscopy guided laser ablation, whereas 2 patients developed peristomal granulations which were also ablated using diode laser. All 4 patients were without complication on long-term follow-up (Table 3).

**Table 5:** Result in terms of airway status and voice clarity after decannulation of Montgomery T-tube.

Serial. n°	Airway status			N° of patients
	Good	Fair	Poor	
1	12	4	3	19

	Voice clarity			
	Good	Fair	Poor	
2	10	5	4	19

In all 16 patients out of 19, the Montgomery T-tube was put for 1 complete year, whereas in 3 patients it was kept for less than 1 complete year. Three patients returned with respiratory distress and required re-tracheostomy with reinsertion of Montgomery tube later on, with burying of vertical limb permanently beneath the skin. These 3 patients were those in which Montgomery T-tube had been removed before the completion of one year.

No granulation was seen near the vocal cords, as during insertion it was ensured that the upper limb of the Montgomery T-Tube does not come out of the vocal cords or touch the undersurface of vocal cords.

## Discussion

Laryngotracheal trauma is reported as second to intracranial injury as the most common cause of death among patients with head and neck trauma.^14^ A blunt or penetrating laryngotracheal injury can result in acute airway obstruction and death at the scene of an accident or crime if not attended with high suspicion in case of multiple trauma patients.

The larynx is shielded inferiorly by the sternum, superiorly by the mandible, posteriorly by the cervical spine, and laterally by the sternocleidomastoid muscles. The laryngeal complex is also supported by muscular and tendinous attachments, which can deflect trauma in all directions except posteriorly. The muscular attachments disperse most of the external forces during impact. This is the reason why laryngeal injuries are infrequent and also the reason why a laryngeal injury is very commonly ignored or overlooked in a polytrauma patient.^15^, ^16^, ^17^

Successful treatment of laryngotracheal trauma requires the incorporation of clinical, physical, and radiographic data for classification and grading of trauma. Management begins with an assessment of the mechanism of injury, the level of injury, and the severity of injury.^18^

The two main classifications of trauma are blunt and penetrating. The most common cause of blunt laryngotracheal trauma is motor vehicle accidents. In most accidents, the driver is pushed against the steering wheel or windshield with the neck extended. This can result in thyroid cartilage fracture, mucosal disruption, edema, arytenoid dislocation, and/or torn laryngeal ligaments.^17^, ^19^ Fortunately, the incidence of blunt laryngotracheal injuries associated with automobile accidents is declining as a result of improved dashboard designs, passenger restraints, air bags, and other safety devices.^20^ Other etiologic considerations in blunt trauma are sports injuries and acts of violence.^21^, ^22^

Presenting symptoms include dyspnea, dysphonia, neck pain, dysphagia, odynophagia, and hemoptysis. The two most common are respiratory distress and dysphonia.^23^ Physical findings include subcutaneous emphysema, tenderness, edema, hematoma, ecchymosis, and distortion or loss of laryngeal landmarks.^24^

Penetrating trauma, its causes and management are beyond the realm of this paper, so will not be discussed.

Radiological assessment with or without Doppler, laryngoscopy, and surgical exploration may be indicated, depending on the nature of the clinical signs and symptoms. Injury to the throat can also be classified according to the anatomic level as either hypopharyngeal, supraglottic, glottic, subglottic, and tracheal. Multiple anatomic levels may be involved in laryngotracheal trauma, based on which we have divided the injuries in our study in 4 categories.

Injuries can be also clinically classified according to Schaefer Fuhrman classification into five types according to the degree and extent of the patient's presenting symptoms and signs.^20^, ^25^

The first priority in any trauma patient is to secure an adequate airway. However, when dealing with laryngotracheal trauma, the initial airway securing method is controversial. Schaefer has stated that intubation following laryngotracheal trauma is hazardous.^9^ However, the American College of Surgeons recommends at least one attempt at intubation, and if it fails only then tracheostomy should be performed.^8^

If the airway is determined to be unstable, we preferred an awake tracheostomy over intubation to avoid further injury to larynx and its supporting structures.^26^ On the other hand patients with major injuries invariably needed tracheostomy and/or laryngeal repair. Timing of surgery showed significant correlation with the outcome in terms of voice and airway. The importance of early operative management has been stressed by several authors recently.^10^, ^11^, ^12^

However, others in the past have recommended delay following injury to enable any edema to resolve.^13^ The distinction between major and minor trauma categories should be made within the first 24 h after trauma. Early surgical intervention is recommended for all major injuries to ensure a good outcome.^26^

In cases where injury involves the glottis/subglottis, or trachea, stents are a plausible means of providing a permanent or temporary airway opening.^27^ Various endotracheal tube types like Montgomery T-tube, Aboul-ker, Dumon and Cotton Lorenz exist. Difficulties in application of expandable metallic stents make these stents not suitable for relief of airway obstruction, and migration of the stent in the airway is a disadvantage of the silicon Dumon stents. The silicon rubber T-tube developed by Montgomery in 1964 has proven widely beneficial in Laryngotracheal surgery. The T-tube has three legs of different diameters and lengths. The external opening can be left open for ventilation and cleaning but can also be closed for voice and ventilation if the lid is closed. Caretta et al. conducted a 158 case study which found that the Montgomery T-tube could be used primarily or complementary to surgical treatment and can be an effective alternative when other stents are unsuccessful.^28^ Thus, we use primarily the Montgomery T-tube in our institute when stenting is desired.

The T-tube is generally well-tolerated. It can be left in situ for years but sometimes must be changed due to infection, granulation, etc. Complications of Montgomery T-tube insertion include infection, granulation at the proximal or distal end of the tube or at the end contacting the skin, granulation tissue on the undersurface of the cords, where the upper end of Montgomery T-tube may sometimes touch, and bacterial colonization or crust formation in the lumen. There is also a risk of tracheomalacia in areas in contact with the tube edges.^29^

The best treatment to prevent laryngotracheal stenosis post-laryngotracheal trauma is laryngotracheal repair and stenting. For this, a tracheal stent is needed, which could be left in the airway for a long time with minimum complications. Such an ideal stent should be rigid enough to resist external pressure, but somehow be soft and flexible too, so that it is minimally irritating to the laryngotracheal mucosa. On the other hand, it should stay fixed in its place in order to not be dislodged by coughing; although it ought to be easily removed when the time of surgery is due or in emergency situations. Due to these advantages, as well as the near-normal phonation that it provides, we prefer to stent our patients with Montgomery T-tubes rather than tracheostomy tubes, Dumon silicone stents, polyflex stents or expandable metallic stents. Another unique advantage of T-tubes is that they are the only stents that could be used for the strictures at the level of the vocal cords and in the subglottic region. On the other hand, the only disadvantage that was noted in our patients was its occlusion by dried mucus and sputum.^30^

## Conclusion

According to our study we suggest that patients with acute blunt laryngotracheal trauma should be managed following the protocol as mentioned in our study, and we strongly emphasize that the Montgomery T-tube should be left for at least 1 complete year in the airway as it results in negligible chances of post-traumatic stenosis of airway later.

## Conflicts of interest

The authors declare no conflicts of interest.
